# Melanocytes from Patients Affected by Ullrich Congenital Muscular Dystrophy and Bethlem Myopathy have Dysfunctional Mitochondria That Can be Rescued with Cyclophilin Inhibitors

**DOI:** 10.3389/fnagi.2014.00324

**Published:** 2014-11-20

**Authors:** Alessandra Zulian, Francesca Tagliavini, Erika Rizzo, Camilla Pellegrini, Francesca Sardone, Nicoletta Zini, Nadir Mario Maraldi, Spartaco Santi, Cesare Faldini, Luciano Merlini, Valeria Petronilli, Paolo Bernardi, Patrizia Sabatelli

**Affiliations:** ^1^Department of Biomedical Sciences, University of Padova, Padova, Italy; ^2^CNR Neuroscience Institute, Padova, Italy; ^3^CNR National Research Council of Italy, Institute of Molecular Genetics, Bologna, Italy; ^4^SC Laboratory of Musculoskeletal Cell Biology, IOR, Bologna, Italy; ^5^Rizzoli Orthopaedic Institute, University of Bologna, Bologna, Italy

**Keywords:** collagen VI, muscular dystrophy, mitochondria, melanocytes, permeability transition, cyclophilin inhibitors

## Abstract

Ullrich congenital muscular dystrophy and Bethlem myopathy are caused by mutations in collagen VI (ColVI) genes, which encode an extracellular matrix protein; yet, mitochondria play a major role in disease pathogenesis through a short circuit caused by inappropriate opening of the permeability transition pore, a high-conductance channel, which causes a shortage in ATP production. We find that melanocytes do not produce ColVI yet they bind it at the cell surface, suggesting that this protein may play a trophic role and that its absence may cause lesions similar to those seen in skeletal muscle. We show that mitochondria in melanocytes of Ullrich congenital muscular dystrophy and Bethlem myopathy patients display increased size, reduced matrix density, and disrupted cristae, findings that suggest a functional impairment. In keeping with this hypothesis, mitochondria (i) underwent anomalous depolarization after inhibition of the F-ATP synthase with oligomycin, and (ii) displayed decreased respiratory reserve capacity. The non-immunosuppressive cyclophilin inhibitor NIM811 prevented mitochondrial depolarization in response to oligomycin in melanocytes from both Ullrich congenital muscular dystrophy and Bethlem myopathy patients, and partially restored the respiratory reserve of melanocytes from one Bethlem myopathy patient. These results match our recent findings on melanocytes from patients affected by Duchenne muscular dystrophy (Pellegrini et al., 2013), and suggest that skin biopsies may represent a minimally invasive tool to investigate mitochondrial dysfunction and to evaluate drug efficacy in ColVI-related myopathies and possibly in other muscle wasting conditions like aging sarcopenia.

## Introduction

Collagen VI (ColVI) is an extracellular matrix (ECM) protein that forms a complex microfibrillar network; it is present in several organs including skeletal muscle, where it is localized just outside the basement membrane (Kuo et al., 1997). ColVI is constituted by three chains (α1, α2, and α3) encoded by different genes (*COL6A1*, *COL6A2*, and *COL6A3*, respectively). Three additional ColVI chains have recently been identified (α4, α5, α6), which are similar to α3 but display a more restricted tissue distribution (Gara et al., 2008). Deficiency of ColVI due to mutations in *COL6A1*, *COL6A2*, or *COL6A3* gives rise to three main muscle disorders, Ullrich congenital muscular dystrophy (UCMD, MIM #254090) (Ullrich, 1930; Camacho Vanegas et al., 2001), Bethlem myopathy (BM, MIM #158810) (Bethlem and Wijngaarden, 1976), and myosclerosis myopathy (MIM #255600) (Merlini et al., 2008b). UCMD is a severe disorder characterized by congenital muscle weakness with axial and proximal joint contractures and coexisting distal joint hypermobility (Bertini and Pepe, 2002). BM is characterized by slowly progressive axial and proximal muscle weakness with finger flexion contractures (Merlini et al., 1994). Myosclerosis myopathy is a recessive disorder characterized by progressive contractures affecting all joints (Merlini et al., 2008b). However, it should be noted that the clinical features of ColVI muscular dystrophy can be extremely heterogenous, ranging from mild to severe myopathy with progressive muscular dystrophy (Jöbsis et al., 1999). Consistent with the idea that these disorders represent a clinical continuum, about 70 different mutations of the *COL6* genes have so far been described in ColVI myopathies (Pepe et al., 2002; Lampe and Bushby, 2005). Patients affected by ColVI muscular dystrophies frequently display skin alterations. Patients with the UCMD phenotype usually present follicular hyperkeratosis over the extensor surfaces of upper and lower limbs, soft velvety skin on the palms and soles, and tendency to develop keloids or “cigarette paper” scars, skin features that may be present also in BM patients (Lampe and Bushby, 2005). Although the mechanism linking ColVI deficiency to skin lesions has not been established, it has recently been shown that melanocytes affect fibroblast proliferation and collagen production, contributing to the generation of hypertrophic scars and keloids (Gao et al., 2013).

Collagen VI myopathies share a common pathogenesis linked to deregulation of the mitochondrial permeability transition pore (PTP), an inner membrane high-conductance channel that forms from dimers of the mitochondrial F-ATP synthase under conditions of Ca^2+^ overload and oxidative stress (Bernardi, 2013; Giorgio et al., 2013) and is desensitized by cyclosporin (Cs) A. Oxidative stress is specifically involved in the pathogenesis of myopathy in the *Col6a1^−/−^* mouse model (Menazza et al., 2010; Sorato et al., 2014); and the resulting myofiber damage is amplified by impaired clearance of defective mitochondria (Grumati et al., 2010). PTP-dependent mitochondrial dysfunction appears to be involved also in other forms of muscular dystrophy, including those caused by lack of δ-sarcoglycan and laminin-2 (Millay et al., 2008), as well as of dystrophin (Millay et al., 2008; Reutenauer et al., 2008; Wissing et al., 2010; Pellegrini et al., 2013). These studies generated pharmacological strategies aimed at rescuing the mitochondrial defect through desensitization of the PTP, and encouraging results have been obtained with the use of CsA and its non-immunosuppressive analogs Debio025 and NIM811 in animal models and in a pilot trial in patients (Irwin et al., 2003; Angelin et al., 2007; Merlini et al., 2008a; Tiepolo et al., 2009; Telfer et al., 2010; Zulian et al., 2014).

Translation of the pharmacological strategies tested in animal models to muscular dystrophy patients is particularly complex, and often requires invasive procedures. Cell cultures derived from muscle biopsies can be used for genetic and mechanistic studies, but in the case of ColVI myopathies the disease phenotype is lost after a few passages, a likely result of *in vitro* selection of apoptosis-resistant cells (Sabatelli et al., 2012b). Melanocytes are the pigment-producing cells of the skin, localized to the basal layer of human epidermis. They are polarized cells performing specific functions at the basolateral and apical membranes, which explains the differential composition of the membrane at these sites (Pinon and Wehrle-Haller, 2011). At the basal layer, melanocytes attach to the dermal–epidermal junction (DEJ), a specialized structure with a fundamental role in maintaining attachment of the epidermis to the dermis and providing skin resistance against shearing forces (Santiago-Walker et al., 2009). Melanocytes do express muscle-specific proteins including the mDP427 dystrophin isoform at the interface with the DEJ (Pellegrini et al., 2013). We have recently demonstrated that melanocytes possess properties similar to those of myoblasts from Duchenne muscular dystrophy patients, and represent a promising cellular model to monitor the response of dystrophinopathies to pharmacological treatments (Pellegrini et al., 2013). Here, we have explored whether mitochondrial dysfunction can be detected in melanocytes from UCMD and BM patients, and whether these cells are a potential alternative to the use of muscle-derived cells in the study and therapy of ColVI myopathies.

## Materials and Methods

### Patients

Skin biopsies from two healthy subjects and four ColVI muscular dystrophy patients (two UCMD and two BM) were collected. All patients were previously diagnosed by genetic, and/or histochemical and biochemical analysis. UCMD patient 1 (UCMD1) carried a *COL6A2* homozygous mutation in exon 28 [UCMD-5 patient in Tagliavini et al. (2014)]; UCMD patient 2 (UCMD2) displayed typical UCMD features, severe deficiency of ColVI in muscle biopsies and defective ColVI secretion in cultured skin fibroblasts; BM patients 1 (BM1) and 2 (BM2) carried heterozygous mutations in *COL6A1* and *COL6A3*, respectively [patients BM-5 and BM-6, respectively, in Tagliavini et al. (2014)]. All participants provided written informed consent, and approval was obtained from the Ethics Committee of the Rizzoli Orthopaedic Institute (Bologna, Italy).

### Cell cultures

Skin fragments were cut into small pieces and the epidermis was separated from the dermis after overnight incubation in 0.5% dispase II (Roche) at 4°C. Melanocytes were maintained in M254 culture medium (GIBCO) supplemented with phorbol-12-myristate 13-acetate, transferrin, hydrocortisone, insulin, bovine pituitary extract, basic fibroblast growth factor, and fetal calf serum (HMGS supplement, GIBCO). Melanocyte-fibroblast co-cultures were obtained by plating fibroblasts onto coverslips in DMEM growth medium until they reached 70% confluence; after 1 day melanocytes were seeded onto the fibroblast cultures and grown in M254 medium supplemented with 0.25 mM l-ascorbic acid (Sigma). ColVI-enriched, conditioned medium was obtained by treating confluent skin fibroblast cultures from healthy donors with DMEM medium supplemented with 0.25 mM l-ascorbic acid. After 24 h, the medium was collected, centrifuged at low speed to remove cell debris and diluted 1:1 with M254 medium. Normal melanocytes plated onto coverslips were grown with ColVI-enriched medium for 24 h before immunohistochemical analysis with anti-ColVI antibody (Millipore). To assess the effect of cyclophilin inhibitors on mitochondrial morphology, melanocytes from patients and healthy subjects were treated with 0.8 μM NIM811 diluted in M254 medium for 2 h at 37°C before processing for immunofluorescence and transmission electron microscopy.

### Immunofluorescence analysis

Cultured melanocytes were fixed with methanol at *−*20°C for 7 min, washed with phosphate-buffered saline, and incubated with antibodies against NG2 (Millipore), integrin β1 (Millipore), pMEL-17 (Monosan), ColVI (Millipore), or Tom20 (Santa Cruz). All antibodies were revealed with secondary anti-rabbit or anti-mouse TRITC or FITC-conjugated antibodies (DAKO). Samples were mounted with an anti-fading reagent (Molecular Probes) and analyzed with a A1-R confocal laser microscope (Nikon) equipped with a Nikon Plan Apo TIRF 100×, 1.45 NA objective, and with 405 and 561 nm laser lines to elicit DAPI (blue) and TRITC (red) fluorescence signals. *Z*-stacks were collected at optical resolution of 120 nm/pixel with pinhole diameter set to 1 Airy unit and *z*-step size to 150 nm. All image analyses were performed using NIS-Elements software (Nikon). For mitochondrial morphometric analysis, maximum intensity projections were generated. Analysis was limited to regions of interest at the periphery of cells, where individual mitochondria are readily resolved. Images were thresholded and converted to binary images. Image segmentation was performed on the binary mask, and number and length of discrete features was measured. The statistical significance of the differences between the experimental points was evaluated by Student’s *t*-test.

### Transmission electron microscopy

Skin biopsy fragments and melanocytes grown onto uncoated well plates were fixed with 2.5% glutaraldehyde in 0.1 M cacodylate buffer for 2 h and post-fixed with 1% osmium tetroxide. After dehydration, samples were detached with propylene oxide, embedded in Epon812 epoxy resin, and observed with a Philips EM400 electron microscope operated at 100 kV after cutting ultrathin sections. For rotary shadowing electron microscopy analysis, melanocyte-fibroblast co-cultures were incubated with anti-ColVI antibody diluted 1:25 in culture medium at 37°C for 2 h. After several washes with culture medium, samples were incubated with anti-mouse IgG 5 nm gold-conjugated antibody diluted 1:20 in culture medium for 1 h at 37°C. Negative controls were performed in the absence of primary antibody. After immunolabeling cells were fixed with 2.5% glutaraldehyde in 0.1 M cacodylate buffer and 1% osmium tetroxide, dehydrated in ethanol and critical point-dried. Thereafter, the slides were rotary-shadowed with platinum at 45°C and coated with carbon at 90°C in a Balzers BAF 400D Freeze Fracture as previously described (Zhang et al., 2002).

### Mitochondrial membrane potential

Mitochondrial membrane potential was measured based on the accumulation of tetramethylrhodamine methyl ester (TMRM, Molecular Probes) (Angelin et al., 2007). Primary cultures of melanocytes obtained as described above from healthy donors, UCMD, and BM patients were seeded onto 24-mm-diameter round glass coverslips and grown for 2 days in M254 culture medium with HMGS supplement (GIBCO). The medium was then replaced with serum-free M254 medium supplemented with 10 nM TMRM for 30 min, and cellular fluorescence images were acquired with an Olympus IX71/IX51 inverted microscope, equipped with a xenon light source (75 W) for epifluorescence illumination and with a 12-bit digital cooled CCD camera (Micromax, Princeton Instruments). Data were acquired and analyzed using Cell R Software (Olympus). For detection of fluorescence, 568 ± 25 nm band-pass excitation and 585 nm long-pass emission filter settings were used. Images were collected with exposure time of 100 ms using a 40×, 1.3 NA oil immersion objective (Nikon). The extent of cell and hence mitochondrial loading with potentiometric probes is affected by the activity of the plasma membrane multidrug resistance pump. In order to normalize the loading conditions, in all experiments with TMRM the medium was supplemented with 1.6 μM CsH, which inhibits the multidrug resistance pump but not the PTP (Bernardi et al., 1999). At the end of each experiment, mitochondria were fully depolarized by the addition of 4 μM of the protonophore carbonyl cyanide-*p*-trifluoromethoxyphenyl hydrazone (FCCP). Clusters of several mitochondria were identified as regions of interest, and fields not containing cells were taken as background. Sequential digital images were acquired every 5 min and the average fluorescence intensity of all relevant regions was recorded and stored for subsequent analysis.

### Oxygen consumption rate

Oxygen consumption rate (OCR) was measured with the XF24 Extracellular Flux Analyzer (Seahorse Bioscience, Billerica, MA, USA). Melanocytes were seeded in XF24 cell culture microplates at 6 × 10^4^ cells/well for patient UCMD1 and 13 × 10^4^ cells/well for patients UCMD2 and BM1 (the same cell densities were used for the matched cultures from healthy donors) in 0.2 ml of M254 culture medium with HMGS supplement and incubated at 37°C in 5% CO_2_ for 24 h. Experiments were carried out on confluent monolayers. Assay were initiated by replacing the growth medium in each well with 0.67 ml of serum-free M254 prewarmed at 37°C. Cells were incubated at 37°C for 30 min to allow temperature and pH equilibration. A titration with FCCP was performed for each cell type in order to determine the optimal FCCP concentration (i.e., the concentration that stimulates respiration maximally), which was found to be 1 μM for cells from healthy donors, 0.8 μM for patient UCMD1, 0.4–0.6 μM for patient UCMD2, and 0.4 μM for patient BM1. After an OCR baseline was established, 70 μl of a solution containing oligomycin, FCCP, rotenone, or antimycin A were sequentially added to each well to reach final concentrations of 1 μg/ml oligomycin, FCCP as stated above, and 1 μM for rotenone and antimycin A. Data are expressed as pmol of O_2_ per minute per 6 × 10^4^ (UCMD1) or 13 × 10^4^ (UCMD2, BM1) cells. At the end of each experiment, the medium was removed from each well and the total protein content per well was quantified. Cells were lysed at 4°C in a buffer composed of 140 mM NaCl, 20 mM Tris-HCl pH 7.4, 5 mM EDTA, 10% glycerol, 1% Triton X-100 (0.1 ml/well) in the presence of phosphatase and protease inhibitors (Sigma). Lysates were then cleared by centrifugation at 13,000 × *g* for 30 min at 4°C, and proteins were quantified using a BCA Protein Assay Kit (Thermo Scientific-Pierce).

## Results

### Melanocytes bind collagen VI microfilaments

**Figure 1:**
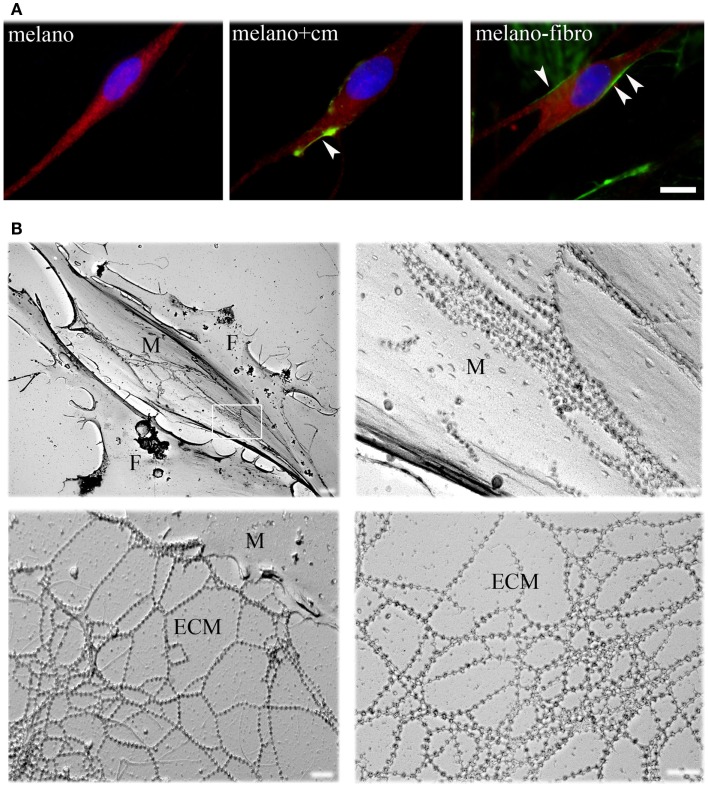
**Immunofluorescence analysis and EM rotary shadowing of melanocyte cultures**. **(A)** Double labeling for ColVI (green) and *p*-MEL17 (red) on melanocyte cultures under basal conditions (melano), after treatment with ColVI-enriched medium conditioned by skin fibroblasts (melano + cm), and of melanocytes co-cultured with skin fibroblasts (melano + fibro); scale bar, 10 μm. **(B)** Rotary shadowing electron microscopy analysis of melanocytes co-cultured with fibroblasts. Melanocytes (M, upper left panel) display the typical bipolar shape compared to the flat morphology of fibroblasts (F). Typical ColVI “beaded” microfilaments, identified by 5 nm colloidal gold particles, are visible both at the melanocytes cell surface (top right panel) and in the ECM (bottom panels). Microfilaments formed parallel rows when attached to the cell surface (upper right panel, which is a magnification of the boxed area in the upper left panel), and web-like structures when deposited in the ECM (lower panels). Scale bar, 400 nm.

To define their interactions with ColVI, we studied pure cultures of melanocytes from healthy donors in the presence of ascorbic acid, which allows hydroxylation of proline and lysine residues and secretion of ColVI *in vitro* (Colombatti and Bonaldo, 1987). ColVI could not be detected in the ECM or inside the cells (Figure 1A, left panel), indicating that melanocytes do not synthesize and secrete this protein. However, when melanocytes were treated with a ColVI-enriched medium obtained from skin fibroblasts (Figure 1A, middle panel) or co-cultured with ColVI-producing skin fibroblasts (Figure 1A, right panel) a clear association of ColVI with the cell surface was detected. Rotary shadowing immunoelectron microscopic analysis with anti-ColVI antibody confirmed the presence of ColVI microfilaments onto the cell membrane of melanocytes (M), which can easily be distinguished from fibroblasts (F) because of their elongated, bipolar shape (Figure 1B, upper left panel). Microfilaments displayed the typical “beaded” pattern with a periodicity of 100 nm, and formed parallel rows when attached to the melanocyte cell surface (Figure 1B, upper right panel), while they formed web-like structures when deposited in the ECM (Figure 1B, lower panels).

### Melanocytes from UCMD and BM display alterations of the mitochondrial network

**Figure 2:**
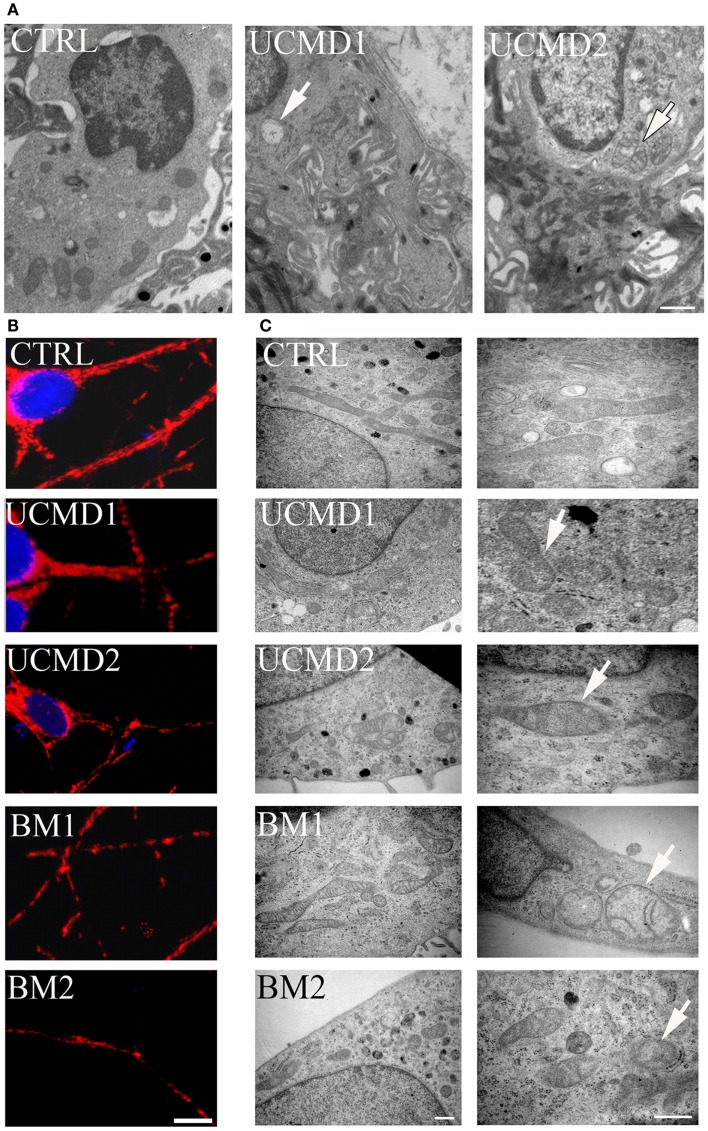
**Morphological analysis of skin biopsies and melanocyte cultures**. **(A)** Ultrastructural analysis of melanocytes in epon-embedded skin biopsies from healthy donor (CTRL) and patients UCMD1 and UCMD2 showing altered mitochondria (arrows) in patient melanocytes; scale bar, 1 μm. **(B)** Confocal imaging of melanocyte cultures from patients UCMD1, UCMD2, BM1, and BM2 and from healthy donor (CTRL) labeled with an anti-Tom20 antibody. Patient melanocytes display a fragmented and discontinuous mitochondrial network compared to the long and branched pattern of the cells from healthy donor; scale bar, 20 μm. **(C)** Ultrastructural analysis of melanocyte cultures from a healthy donor and patients, showing the presence of enlarged mitochondria with a few cristae (arrows) in all patients; scale bar, 500 nm.

Skin biopsies of healthy donors and of UCMD patients were studied by ultrastructural analysis. Mitochondrial changes, including increased size, reduced matrix density, and disrupted cristae consistent with swelling, were frequently found in patients (Figure 2A, arrows in middle and right panels), while mitochondria in melanocytes of healthy controls had the expected features (Figure 2A, left panel). Occurrence of mitochondrial alterations was also detected in melanocyte cultures obtained from both UCMD and BM patients. The mitochondrial reticulum, as monitored by immunofluorescence analysis of the outer membrane protein Tom20, appeared fragmented along the cytoplasmic extensions as indicated by the presence of a punctate labeling pattern (Figure 2B). Ultrastructural analysis showed changes similar to those detected in skin, with mitochondria of irregular size and focal swelling (Figure 2C, arrows).

### Mitochondria of UCMD and BM melanocytes display a latent dysfunction

**Figure 3:**
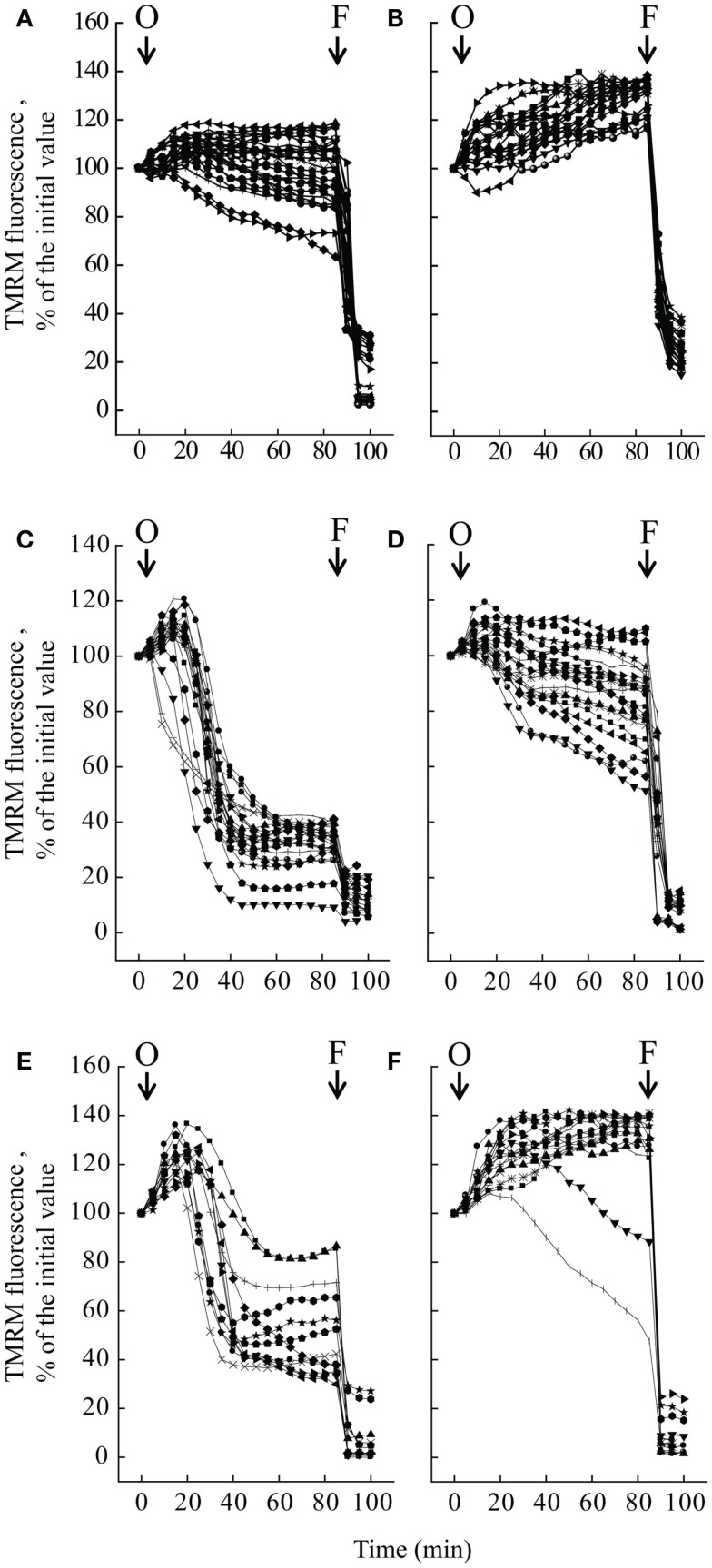
**Effect of oligomycin on mitochondrial membrane potential in melanocytes**. Melanocytes from one healthy donor **(A,B)**, from patient UCMD2 **(C,D)** and from patient BM1 **(E,F)** were loaded with TMRM and studied by epifluorescence microscopy as described (Pellegrini et al., 2013). Where indicated (arrows), 6 μM oligomycin (O) and 4 μM FCCP (F) were added. In the experiments of **(B,D,F)**, cells had been treated with 0.8 μM NIM811 for 30 min before beginning the recordings. Each line reports mitochondrial fluorescence of one individual cell.

**Figure 4:**
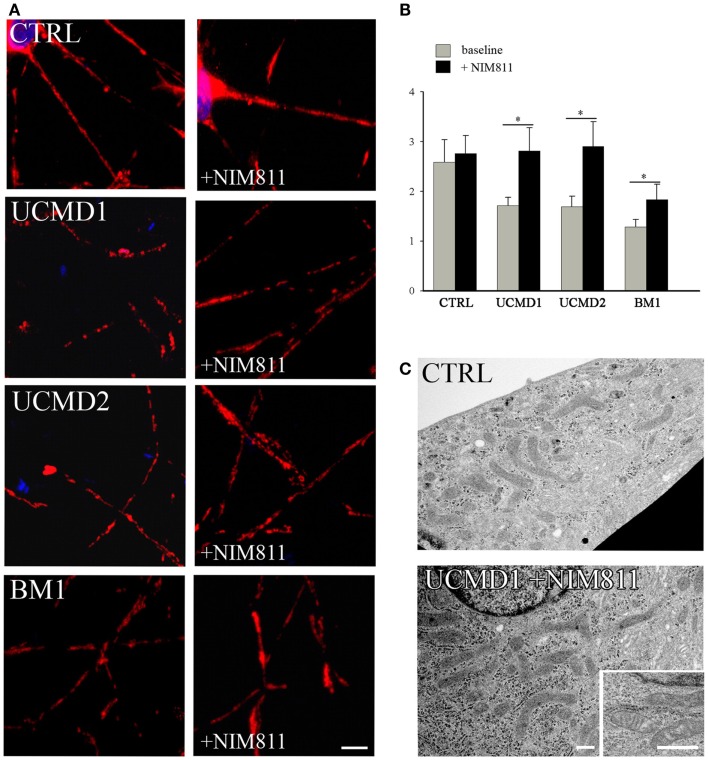
**Effect of NIM811 on mitochondrial morphology**. **(A)** Melanocytes from a healthy donor, and from UCMD1, UCMD2, and BM1 patients were treated with 0.8 μM NIM811 for 2 h and labeled with anti-Tom20 antibody. Scale bar, 5 μm. **(B)** Mitochondrial length (μm) was quantified and is reported as mean ± SEM; *t*-test; *P < 0.05; **(C)** Ultrastructural analysis of melanocytes from a healthy donor (CTRL) and patient UCMD1 (representative of all patients), after treatment with NIM811 (UCMD1 + NIM811). Scale bar, 300 nm.

We studied mitochondrial function in primary cultures of melanocytes obtained from one healthy donor and from patients UCMD2 and BM1. Irrespective of the presence of NIM811, addition of oligomycin to melanocytes from healthy donor was followed by an initial fluorescence increase (i.e., the expected hyperpolarization), which was readily followed by depolarization (i.e., probe release with decreased mitochondrial fluorescence) upon addition of the protonophore FCCP (Figures 3A,B). At variance from melanocytes from healthy donor, upon addition of oligomycin melanocytes from patients UCMD2 (Figure 3C) and BM1 (Figure 3E) underwent rapid mitochondrial depolarization after the initial hyperpolarization. Consistent with a key role of the PTP in onset of depolarization, the response to oligomycin was normalized by the cyclophilin inhibitor NIM811, a CsA analog devoid of immunosuppressive activity that desensitizes the PTP without inhibiting calcineurin (Zulian et al., 2014) (Figures 3D,F). Treatment with NIM811 normalized mitochondrial morphology of UCMD1, UCMD2, and BM1, improving the mitochondrial reticulum extension as indicated by increased mitochondrial length (Figures 4A,B). Ultrastructural analysis confirmed that treatment with NIM811 restored mitochondrial matrix density and cristae organization in patient melanocytes (Figure 4C), without affecting mitochondria of melanocytes from the healthy patient (Figures 4B,C). It should be noted that NIM811 inhibits all CyP isoforms, and that an isoform-selective inhibitor is only available for CyPA (Daum et al., 2009); yet, since mitochondria lack other Cs-binding proteins (Nicolli et al., 1996), we conclude that the mitochondrial effects of NIM811 are mediated by inhibition of CyPD. Taken together, these results demonstrate that UCMD and BM melanocytes display the same PTP-dependent latent mitochondrial dysfunction previously identified in primary muscle-derived cell cultures from *Col6a1^−/−^* mice (Irwin et al., 2003), UCMD and BM patients (Angelin et al., 2007), and zebrafish with ColVI myopathy (Telfer et al., 2010; Zulian et al., 2014).

### Mitochondrial respiratory reserve capacity is decreased in melanocyte cultures from UCMD and BM patients

**Figure 5:**
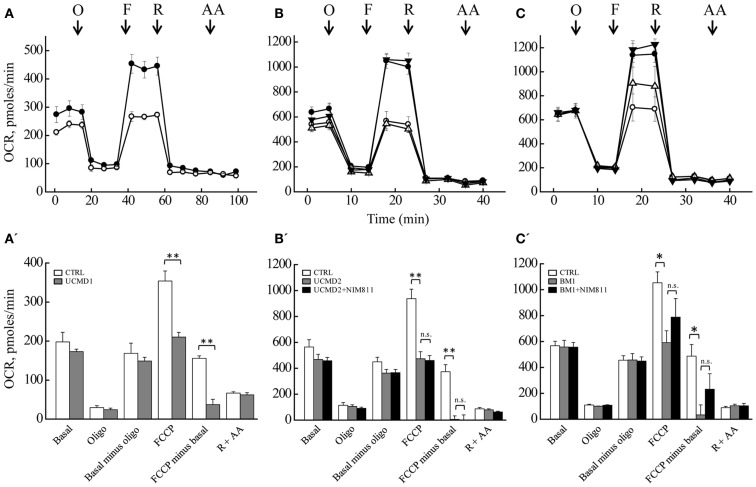
**Oxygen consumption rate of melanocytes**. OCR of melanocytes from a healthy donor (closed circles in all panels), of patients (open circles in all panels) UCMD1 **(A)**, UCMD2 **(B)**, and BM1 **(C)** was measured in 24-well Seahorse plates [60,000 cells/well for **(A)** and 130,000 cells/well for **(B,C)**]. Where indicated (arrows) 1 μg/ml oligomycin (O), FCCP as detailed in Section “Materials and Methods” (F), 1 μM rotenone (R), and 1 μM antimycin A (AA) were added. In **(B,C)**, triangles report respiration of melanocytes from healthy donor (closed symbols) or UCMD2 and BM1 patients (open symbols) treated with 0.8 μM NIM811 1 h before beginning the recording. Data in each panel are mean of at least four independent determinations in triplicate ± SEM. **(A’–C’)** OCR before (Basal) and after the sequential addition of oligomycin (Oligo), FCCP, rotenone, and antimycin A. Data were analyzed with ANOVA. *P < 0.05; **P < 0.005; n.s., not significant.

We measured the OCR of primary cultures of melanocytes from UCMD and BM patients with the sensitive Seahorse technology (Wu et al., 2007). Basal respiration largely reflects mitochondrial oxygen consumption, which in aerobic, non-transformed cells fulfills the needs for ATP synthesis; addition of oligomycin inhibits the F-ATP synthase, and therefore, “removes” the fraction of respiration linked to ATP synthesis. Inspection of the response of melanocytes from healthy donors (Figures 5A–C, closed circles) reveals that a large fraction of the basal OCR is linked to ATP synthesis, as shown by the inhibitory effect of oligomycin (residual oxygen consumption is non-mitochondrial, see below). Melanocytes could be stimulated well above the basal respiratory level by addition of the protonophore FCCP, which stimulates respiration maximally, demonstrating that they have a large respiratory reserve (the difference between OCR after the addition of FCCP and basal OCR). Addition of rotenone (selective inhibitor of respiratory complex I) almost completely inhibited the OCR, no further decrease being seen with the addition of antimycin A (selective inhibitor of complex III). Finally, NIM811 had no effect in the response of melanocytes from healthy donors to oligomycin, FCCP, rotenone, and antimycin A (Figures 5B,C closed triangles). Melanocytes from patients UCMD1, UCMD2, and BM1 displayed a marked decrease of the respiratory reserve, which became apparent after the addition of FCCP (open circles in Figures 5A–C, respectively). In patient BM1, maximal respiration was partially restored by NIM811 but the effect was not statistically significant (Figure 5C). Thus, assessing whether the lower respiratory activity of melanocytes from patients depends on PTP opening will require further work. We also tested the effect of FCCP on basal respiration (i.e., in the absence of oligomycin) with identical results (data not shown). The statistical analysis of these experiments is presented in Figures 5A’–C’. Resting ATP levels were not altered in melanocytes from ColVI patients (results not shown), as also observed in myoblast cultures (Angelin et al., 2008). These findings, which match the maintenance of a normal mitochondrial membrane potential under resting conditions, are consistent with a latent rather than overt mitochondrial dysfunction in cultured melanocytes from the patients.

## Discussion

Epidermal melanocytes interact with the underlying ECM of derma via the DEJ, a specialized adhesion structure consisting of ECM components that include ColVI (Sabatelli et al., 2011). Adhesion of melanocytes to the DEJ involves components common to the muscle cell sarcolemma. Like muscle cells, melanocytes express mDp427 dystrophin (Pellegrini et al., 2013), which bridges the cytoskeleton and the ECM through α, β dystroglycan (Herzog et al., 2004) and the α2 chain of laminin (Sewry et al., 1996). In addition, the DEJ basement membrane is enriched in ColVI, which includes the α5 and α6 chains (Sabatelli et al., 2011) as also recently shown for the endomysium of human muscle (Sabatelli et al., 2012a). ColVI microfilament organization is influenced by the interactions of ColVI with its binding partners (Wiberg et al., 2002). Our findings demonstrate that, like myoblasts (Zou et al., 2008; Sabatelli et al., 2012b), melanocytes do not produce ColVI, yet they bind the protein at the cell surface (Kawahara et al., 2008). Melanocytes do express NG2 proteoglycan (results not shown), which acts as a ColVI receptor (Campoli et al., 2010) together with integrins (Pfaff et al., 1993; Zambruno et al., 1993). The pattern of melanocyte surface labeling with ColVI microfibrils is consistent with receptor-mediated interactions. Interestingly, lack of ColVI affects melanocyte mitochondria in the same way as it affects mitochondria in skeletal muscle, suggesting that this ECM protein exerts a trophic role in the skin as well, consistent with the lesions detected in UCMD and BM patients (Lampe and Bushby, 2005). Indeed, UCMD and BM melanocytes display the same PTP-dependent latent mitochondrial dysfunction previously identified in primary muscle-derived cell cultures from *Col6a1^−/−^* mice (Irwin et al., 2003), UCMD and BM patients (Angelin et al., 2007), and zebrafish with ColVI myopathy (Zulian et al., 2014) that manifests itself as an oligomycin-induced depolarization. We have interpreted oligomycin-induced depolarization as a secondary event following mitochondrial Ca^2+^ overload; this would be a consequence of decreased activity of ATP-dependent Ca^2+^ pumps, resulting in cytosolic Ca^2+^ overload because of decreased Ca^2+^ extrusion at the plasma membrane and decreased Ca^2+^ uptake from the endo-sarcoplasmic reticulum (Angelin et al., 2008).

The reason why maximal respiration is decreased in melanocytes from ColVI muscular dystrophy patients is not easy to dissect in intact cells, but prominent possibilities are increased cytochrome *c* release through both PTP-dependent and -independent mechanisms (Clerc et al., 2012), and PTP-dependent pyridine nucleotide depletion (Vinogradov et al., 1972; Di Lisa et al., 2001). Short openings of the PTP that cannot be detected by TMRM redistribution do take place in isolated mitochondria (Hüser et al., 1998; Hüser and Blatter, 1999) and have also been documented *in situ* (Petronilli et al., 1999). The PTP open time can be increased by Ca^2+^ overload synergistically with arachidonic acid produced by activation of cPLA_2_ (Petronilli et al., 2001; Scorrano et al., 2001; Penzo et al., 2004) leading to cytochrome *c* release (Petronilli et al., 2001), depletion of matrix pyridine nucleotides (Di Lisa et al., 2001), and respiratory inhibition (Vinogradov et al., 1972). Since we found ultrastructural and functional alterations of mitochondria compatible with increased PTP opening in melanocytes from UCMD and BM patients, we suspect that PTP opening is responsible for decreased maximal respiratory capacity as well. Some support for this interpretation comes from the partial restoration of respiration in patient BM1 after treatment with the cyclophilin inhibitor NIM811, which desensitizes the PTP.

The present results show that skin melanocytes from UCMD and BM patients faithfully reproduce the mitochondrial dysfunction and ultrastructural alterations that characterize myoblasts and myofibers from the same patients (Merlini and Bernardi, 2008). These findings may also help to understand the pathogenesis of the skin lesions in patients affected by ColVI muscular dystrophies, and closely match recent results on melanocytes derived from a patient affected by Duchenne muscular dystrophy (Pellegrini et al., 2013). Taken together, our results suggest that melanocytes can become a useful tool to study and monitor other muscle diseases as well, and that skin biopsies may represent a convenient and minimally invasive alternative procedure to monitor muscle genetic diseases and to assess potential therapies *ex vivo*.

## Conflict of Interest Statement

The study was supported in part by a Research Grant from Novartis Pharma AG (Basel).

## Abbreviations

BM, Bethlem myopathy; ColVI, collagen VI; Cs, cyclosporin; DEJ, dermal–epidermal junction; ECM, extracellular matrix; FCCP, carbonylcyanide-*p*-trifluoromethoxyphenyl hydrazone; OCR, oxygen consumption rate; PTP, mitochondrial permeability transition pore; TMRM, tetramethylrhodamine methyl ester; UCMD, Ullrich congenital muscular dystrophy.

## References

[B1] Angelin A. Bonaldo P. Bernardi P. (2008). Altered threshold of the mitochondrial permeability transition pore in Ullrich congenital muscular dystrophy. Biochim. Biophys. Acta 1777, 893–896. 10.1016/j.bbabio.2008.03.026 18435905

[B2] Angelin A. Tiepolo T. Sabatelli P. Grumati P. Bergamin N. Golfieri C. (2007). Mitochondrial dysfunction in the pathogenesis of Ullrich congenital muscular dystrophy and prospective therapy with cyclosporins. Proc. Natl. Acad. Sci. U.S.A. 104, 991–996. 10.1073/pnas.0610270104 17215366 PMC1783427

[B3] Bernardi P. (2013). The mitochondrial permeability transition pore: a mystery solved? Front. Physiol. 4:95. 10.3389/fphys.2013.00095 23675351 PMC3650560

[B4] Bernardi P. Scorrano L. Colonna R. Petronilli V. Di Lisa F. (1999). Mitochondria and cell death. Mechanistic aspects and methodological issues. Eur. J. Biochem. 264, 687–701. 10.1046/j.1432-1327.1999.00725.x 10491114

[B5] Bertini E. Pepe G. (2002). Collagen type VI and related disorders: Bethlem myopathy and Ullrich scleroatonic muscular dystrophy. Eur. J. Paediatr. Neurol. 6, 193–198 10.1053/ejpn.2002.0593 12374585

[B6] Bethlem J. Wijngaarden G. K. (1976). Benign myopathy, with autosomal dominant inheritance. A report on three pedigrees. Brain 99, 91–100. 10.1093/brain/99.1.91 963533

[B7] Camacho Vanegas O. Bertini E. Zhang R. Z. Petrini S. Minosse C. Sabatelli P. (2001). Ullrich scleroatonic muscular dystrophy is caused by recessive mutations in collagen type VI. Proc. Natl. Acad. Sci. U.S.A. 98, 7516–7521. 10.1073/pnas.121027598 11381124 PMC34700

[B8] Campoli M. Ferrone S. Wang X. (2010). Functional and clinical relevance of chondroitin sulfate proteoglycan 4. Adv. Cancer Res. 109, 73–121. 10.1016/B978-0-12-380890-5.00003-X 21070915

[B9] Clerc P. Carey G. B. Mehrabian Z. Wei M. Hwang H. Girnun G. D. (2012). Rapid detection of an ABT-737-sensitive primed for death state in cells using microplate-based respirometry. PLoS ONE 7:e42487. 10.1371/journal.pone.0042487 22880001 PMC3411749

[B10] Colombatti A. Bonaldo P. (1987). Biosynthesis of chick type VI collagen. II. Processing and secretion in fibroblasts and smooth muscle cells. J. Biol. Chem. 262, 14461–14466. 3667584

[B11] Daum S. Schumann M. Mathea S. Aumuller T. Balsley M. A. Constant S. L. (2009). Isoform-specific inhibition of cyclophilins. Biochemistry 48, 6268–6277. 10.1021/bi9007287 19480458 PMC2753677

[B12] Di Lisa F. Menabò R. Canton M. Barile M. Bernardi P. (2001). Opening of the mitochondrial permeability transition pore causes depletion of mitochondrial and cytosolic NAD^+^ and is a causative event in the death of myocytes in postischemic reperfusion of the heart. J. Biol. Chem. 276, 2571–2575. 10.1074/jbc.M006825200 11073947

[B13] Gao F. L. Jin R. Zhang L. Zhang Y. G. (2013). The contribution of melanocytes to pathological scar formation during wound healing. Int. J. Clin. Exp. Med. 6, 609–613. 23936604 PMC3731197

[B14] Gara S. K. Grumati P. Urciuolo A. Bonaldo P. Kobbe B. Koch M. (2008). Three novel collagen VI chains with high homology to the alpha3 chain. J. Biol. Chem. 283, 10658–10670. 10.1074/jbc.M709540200 18276594

[B15] Giorgio V. von Stockum S. Antoniel M. Fabbro A. Fogolari F. Forte M. (2013). Dimers of mitochondrial ATP synthase form the permeability transition pore. Proc. Natl. Acad. Sci. U.S.A. 110, 5887–5892. 10.1073/pnas.1217823110 23530243 PMC3625323

[B16] Grumati P. Coletto L. Sabatelli P. Cescon M. Angelin A. Bertaggia E. (2010). Autophagy is defective in collagen VI muscular dystrophies, and its reactivation rescues myofiber degeneration. Nat. Med. 16, 1313–1320. 10.1038/nm.2247 21037586

[B17] Herzog C. Has C. Franzke C. W. Echtermeyer F. G. Schlötzer-Schrehardt U. Kröger S. (2004). Dystroglycan in skin and cutaneous cells: beta-subunit is shed from the cell surface. J. Invest. Dermatol. 122, 1372–1380. 10.1111/j.0022-202X.2004.22605.x 15175026

[B18] Hüser J. Blatter L. A. (1999). Fluctuations in mitochondrial membrane potential caused by repetitive gating of the permeability transition pore. Biochem. J. 343(Pt 2), 311–317. 10.1042/0264-6021:3430311 10510294 PMC1220555

[B19] Hüser J. Rechenmacher C. E. Blatter L. A. (1998). Imaging the permeability pore transition in single mitochondria. Biophys. J. 74, 2129–2137. 10.1016/S0006-3495(98)77920-2 9545072 PMC1299554

[B20] Irwin W. A. Bergamin N. Sabatelli P. Reggiani C. Megighian A. Merlini L. (2003). Mitochondrial dysfunction and apoptosis in myopathic mice with collagen VI deficiency. Nat. Genet. 35, 267–271 10.1038/ng1270 14625552

[B21] Jöbsis G. J. Boers J. M. Barth P. G. de Visser M. (1999). Bethlem myopathy: a slowly progressive congenital muscular dystrophy with contractures. Brain 122(Pt 4), 649–655. 10.1093/brain/122.4.649 10219778

[B22] Kawahara G. Ogawa M. Okada M. Malicdan M. C. Goto Y. Hayashi Y. K. (2008). Diminished binding of mutated collagen VI to the extracellular matrix surrounding myocytes. Muscle Nerve 38, 1192–1195. 10.1002/mus.21030 18642359

[B23] Kuo H. J. Maslen C. L. Keene D. R. Glanville R. W. (1997). Type VI collagen anchors endothelial basement membranes by interacting with type IV collagen. J. Biol. Chem. 272, 26522–26529. 10.1074/jbc.272.42.26522 9334230

[B24] Lampe A. K. Bushby K. M. (2005). Collagen VI related muscle disorders. J. Med. Genet. 42, 673–685. 10.1136/jmg.2002.002311 16141002 PMC1736127

[B25] Menazza S. Blaauw B. Tiepolo T. Toniolo L. Braghetta P. Spolaore B. (2010). Oxidative stress by monoamine oxidases is causally involved in myofiber damage in muscular dystrophy. Hum. Mol. Genet. 19, 4207–4215. 10.1093/hmg/ddq339 20716577

[B26] Merlini L. Angelin A. Tiepolo T. Braghetta P. Sabatelli P. Zamparelli A. (2008a). Cyclosporin A corrects mitochondrial dysfunction and muscle apoptosis in patients with collagen VI myopathies. Proc. Natl. Acad. Sci. U.S.A. 105, 5225–5229. 10.1073/pnas.0800962105 18362356 PMC2278179

[B27] Merlini L. Martoni E. Grumati P. Sabatelli P. Squarzoni S. Urciuolo A. (2008b). Autosomal recessive myosclerosis myopathy is a collagen VI disorder. Neurology 71, 1245–1253. 10.1212/01.wnl.0000327611.01687.5e 18852439

[B28] Merlini L. Bernardi P. (2008). Therapy of collagen VI-related myopathies (Bethlem and Ullrich). Neurotherapeutics 5, 613–618. 10.1016/j.nurt.2008.08.004 19019314 PMC4514708

[B29] Merlini L. Morandi L. Granata C. Ballestrazzi A. (1994). Bethlem myopathy: early-onset benign autosomal dominant myopathy with contractures. Description of two new families. Neuromuscul. Disord. 4, 503–511. 10.1016/0960-8966(94)90091-4 7881296

[B30] Millay D. P. Sargent M. A. Osinska H. Baines C. P. Barton E. R. Vuagniaux G. (2008). Genetic and pharmacologic inhibition of mitochondrial-dependent necrosis attenuates muscular dystrophy. Nat. Med. 14, 442–447. 10.1038/nm1736 18345011 PMC2655270

[B31] Nicolli A. Basso E. Petronilli V. Wenger R. M. Bernardi P. (1996). Interactions of cyclophilin with the mitochondrial inner membrane and regulation of the permeability transition pore, a cyclosporin A-sensitive channel. J. Biol. Chem. 271, 2185–2192. 10.1074/jbc.271.4.2185 8567677

[B32] Pellegrini C. Zulian A. Gualandi F. Manzati E. Merlini L. Michelini M. E. (2013). Melanocytes-A novel tool to study mitochondrial dysfunction in Duchenne muscular dystrophy. J. Cell Physiol. 228, 1323–1331. 10.1002/jcp.24290 23169061 PMC3601437

[B33] Penzo D. Petronilli V. Angelin A. Cusan C. Colonna R. Scorrano L. (2004). Arachidonic acid released by phospholipase A2 activation triggers Ca^2+^-dependent apoptosis through the mitochondrial pathway. J. Biol. Chem. 279, 25219–25225. 10.1074/jbc.M310381200 15070903

[B34] Pepe G. Bertini E. Bonaldo P. Bushby K. Giusti B. de Visser M. (2002). Bethlem myopathy (BETHLEM) and Ullrich scleroatonic muscular dystrophy: 100th ENMC international workshop, 23-24 November 2001, Naarden, The Netherlands. Neuromuscul. Disord. 12, 984–993 10.1016/S0960-8966(02)00139-6 12467756

[B35] Petronilli V. Miotto G. Canton M. Brini M. Colonna R. Bernardi P. (1999). Transient and long-lasting openings of the mitochondrial permeability transition pore can be monitored directly in intact cells by changes in mitochondrial calcein fluorescence. Biophys. J. 76, 725–734. 10.1016/S0006-3495(99)77239-5 9929477 PMC1300077

[B36] Petronilli V. Penzo D. Scorrano L. Bernardi P. Di Lisa F. (2001). The mitochondrial permeability transition, release of cytochrome c and cell death. Correlation with the duration of pore openings in situ. J. Biol. Chem. 276, 12030–12034. 10.1074/jbc.M010604200 11134038

[B37] Pfaff M. Aumailley M. Specks U. Knolle J. Zerwes H. G. Timpl R. (1993). Integrin and Arg-Gly-Asp dependence of cell adhesion to the native and unfolded triple helix of collagen type VI. Exp. Cell Res. 206, 167–176. 10.1006/excr.1993.1134 8387021

[B38] Pinon P. Wehrle-Haller B. (2011). Integrins: versatile receptors controlling melanocyte adhesion, migration and proliferation. Pigment Cell Melanoma Res. 24, 282–294. 10.1111/j.1755-148X.2010.00806.x 21087420

[B39] Reutenauer J. Dorchies O. M. Patthey-Vuadens O. Vuagniaux G. Ruegg U. T. (2008). Investigation of Debio 025, a cyclophilin inhibitor, in the dystrophic mdx mouse, a model for Duchenne muscular dystrophy. Br. J. Pharmacol 155, 574–584. 10.1038/bjp.2008.285 18641676 PMC2579666

[B40] Sabatelli P. Gara S. K. Grumati P. Urciuolo A. Gualandi F. Curci R. (2011). Expression of the collagen VI alpha5 and alpha6 chains in normal human skin and in skin of patients with collagen VI-related myopathies. J. Invest. Dermatol. 131, 99–107. 10.1038/jid.2010.284 20882040

[B41] Sabatelli P. Gualandi F. Gara S. K. Grumati P. Zamparelli A. Martoni E. (2012a). Expression of collagen VI alpha5 and alpha6 chains in human muscle and in Duchenne muscular dystrophy-related muscle fibrosis. Matrix Biol. 31, 187–196. 10.1016/j.matbio.2011.12.003 22226732 PMC3315014

[B42] Sabatelli P. Palma E. Angelin A. Squarzoni S. Urciuolo A. Pellegrini C. (2012b). Critical evaluation of the use of cell cultures for inclusion in clinical trials of patients affected by collagen VI myopathies. J. Cell. Physiol. 227, 2927–2935. 10.1002/jcp.23039 21953374 PMC3415679

[B43] Santiago-Walker A. Li L. Haass N. K. Herlyn M. (2009). Melanocytes: from morphology to application. Skin Pharmacol. Physiol. 22, 114–121 10.1159/000178870 19188759

[B44] Scorrano L. Penzo D. Petronilli V. Pagano F. Bernardi P. (2001). Arachidonic acid causes cell death through the mitochondrial permeability transition. Implications for tumor necrosis factor-α apoptotic signaling. J. Biol. Chem. 276, 12035–12040. 10.1074/jbc.M010603200 11134037

[B45] Sewry C. A. Philpot J. Sorokin L. M. Wilson L. A. Naom I. Goodwin F. (1996). Diagnosis of merosin (laminin-2) deficient congenital muscular dystrophy by skin biopsy. Lancet 347, 582–584. 10.1016/S0140-6736(96)91274-X 8596321

[B46] Sorato E. Menazza S. Zulian A. Sabatelli P. Gualandi F. Merlini L. (2014). Monoamine oxidase inhibition prevents mitochondrial dysfunction and apoptosis in myoblasts from patients with collagen VI myopathies. Free Radic. Biol. Med. 75C, 40–47. 10.1016/j.freeradbiomed.2014.07.006 25017965 PMC4180008

[B47] Tagliavini F. Pellegrini C. Sardone F. Squarzoni S. Paulsson M. Wagener R. (2014). Defective collagen VI alpha6 chain expression in the skeletal muscle of patients with collagen VI-related myopathies. Biochim. Biophys. Acta 1842, 1604–1612. 10.1016/j.bbadis.2014.05.033 24907562 PMC4316388

[B48] Telfer W. R. Busta A. S. Bonnemann C. G. Feldman E. L. Dowling J. J. (2010). Zebrafish models of collagen VI-related myopathies. Hum. Mol. Genet. 19, 2433–2444. 10.1093/hmg/ddq126 20338942 PMC2876888

[B49] Tiepolo T. Angelin A. Palma E. Sabatelli P. Merlini L. Nicolosi L. (2009). The cyclophilin inhibitor Debio 025 normalizes mitochondrial function, muscle apoptosis and ultrastructural defects in *Col6a1^-/-^* myopathic mice. Br. J. Pharmacol. 157, 1045–1052. 10.1111/j.1476-5381.2009.00316.x 19519726 PMC2737663

[B50] Ullrich O. (1930). Kongenitale, atonisch-sklerotische Muskeldystrophie, ein weiterer Typus der heredodegenerativen Erkrankungen des neuromuskulaeren Systems. Z. Ges. Neurol. Psychiatr. 126, 171–201 10.1007/BF02864097

[B51] Vinogradov A. Scarpa A. Chance B. (1972). Calcium and pyridine nucleotide interaction in mitochondrial membranes. Arch. Biochem. Biophys. 152, 646–654 10.1016/0003-9861(72)90261-5 4344129

[B52] Wiberg C. Heinegård D. Wenglén C. Timpl R. Mörgelin M. (2002). Biglycan organizes collagen VI into hexagonal-like networks resembling tissue structures. J. Biol. Chem. 277, 49120–49126. 10.1074/jbc.M206891200 12354766

[B53] Wissing E. R. Millay D. P. Vuagniaux G. Molkentin J. D. (2010). Debio-025 is more effective than prednisone in reducing muscular pathology in mdx mice. Neuromuscul. Disord. 20, 753–760. 10.1016/j.nmd.2010.06.016 20637615 PMC2980760

[B54] Wu M. Neilson A. Swift A. L. Moran R. Tamagnine J. Parslow D. (2007). Multiparameter metabolic analysis reveals a close link between attenuated mitochondrial bioenergetic function and enhanced glycolysis dependency in human tumor cells. Am. J. Physiol. Cell Physiol. 292, C125–C136. 10.1152/ajpcell.00247.2006 16971499

[B55] Zambruno G. Marchisio P. C. Melchiori A. Bondanza S. Cancedda R. De Luca M. (1993). Expression of integrin receptors and their role in adhesion, spreading and migration of normal human melanocytes. J. Cell Sci. 105(Pt 1), 179–190. 8360272 10.1242/jcs.105.1.179

[B56] Zhang R. Z. Sabatelli P. Pan T. C. Squarzoni S. Mattioli E. Bertini E. (2002). Effects on collagen VI mRNA stability and microfibrillar assembly of three COL6A2 mutations in two families with Ullrich congenital muscular dystrophy. J. Biol. Chem. 277, 43557–43564. 10.1074/jbc.M207696200 12218063

[B57] Zou Y. Zhang R. Z. Sabatelli P. Chu M. L. Bonnemann C. G. (2008). Muscle interstitial fibroblasts are the main source of collagen VI synthesis in skeletal muscle: implications for congenital muscular dystrophy types Ullrich and Bethlem. J. Neuropathol. Exp. Neurol. 67, 144–154. 10.1097/nen.0b013e3181634ef7 18219255

[B58] Zulian A. Rizzo E. Schiavone M. Palma E. Tagliavini F. Blaauw B. (2014). NIM811, a cyclophilin inhibitor without immunosuppressive activity, is beneficial in collagen VI congenital muscular dystrophy models. Hum. Mol. Genet. 23, 5353–5363. 10.1093/hmg/ddu254 24852368

